# The Dietary Supplemental Effect of Nitroethanol in Comparison with Monensin on Methane Emission, Growth Performance and Carcass Characteristics in Female Lambs

**DOI:** 10.3390/ani11020327

**Published:** 2021-01-28

**Authors:** Zhen-Wei Zhang, Yan-Lu Wang, Yong-Yan Chen, Luo-Tong Zhang, Ying-Jie Zhang, Yue-Qin Liu, Yun-Xia Guo, Hong-Jian Yang

**Affiliations:** 1 State Key Laboratory of Animal Nutrition, College of Animal Science and Technology, China Agricultural University, Beijing 100193, China; qingyibushuo@163.com (Z.-W.Z.); yanluwang@yeah.net (Y.-L.W.); yanz00@foxmail.com (Y.-Y.C.); zhangluotong@agri.gov.cn (L.-T.Z.); 2 Liaocheng Research Institute of Donkey High-Efficiency Breeding and Ecological Feeding, Liaocheng University, Liaocheng 252059, China; 3 College of Animal Science and Technology, Hebei Agricultural University, Baoding 071000, China; zhangyingjie66@126.com (Y.-J.Z.); liuyueqin66@126.com (Y.-Q.L.); 4 College of Life Science, Hebei Agricultural University, Baoding 071000, China; gyx310@163.com

**Keywords:** 2-nitroethanol, monensin, CH_4_ emission, growth performance, carcass characteristics

## Abstract

**Simple Summary:**

The objective of present study was to determine the effects of 2-nitroethanol (NEOH) in comparison with monensin on methane (CH_4_) emission, growth performance and carcass characteristics in female lambs. Both monensin and NEOH are potent CH_4_ inhibitors that can reduce dietary energy loss. The average daily gain (ADG) and feed conversion rate were improved with monensin and NEOH addition, suggesting that NEOH in comparison with monensin presented a similarly lasting beneficial effect on feed efficiency for female lambs. In addition, monensin and NEOH increased the net muscle percentage to carcass weight (*p* = 0.03), and they caused a reduction in feed consumption and feed costs resulting in a higher net revenue and economic efficiency. In summary, NEOH in comparison with monensin presented a greater promoting effect on energy utilization in the female feedlotting lambs by inhibiting rumen methanogenesis more efficiently.

**Abstract:**

This study was conducted to evaluate the dietary supplemental effects of 2-nitroethanol (NEOH) in comparison with monensin on methane (CH_4_) emission, growth performance and carcass characteristics in female lambs. Sixty female, small-tailed Chinese Han lambs (3.5 ± 0.3 month) were randomly allotted into three dietary treatment groups: (1) Control group, a basal control diet, (2) monensin group, the basal diet added with 40 mg/kg monensin, (3) NEOH group, the basal diet added with 277 mg/kg nitroethanol, and the feedlotting trial lasted for 70 days. Although dietary addition of monensin and NEOH did not affect nutrient digestibility of lambs, both monensin and NEOH decreased the calculated CH_4_ production (12.7% vs. 17.4% decrease; *p* < 0.01). In addition, the CH_4_ production represents less dietary energy loss in the monensin and NEOH group than in the control, indicating that monensin and NEOH are potent CH_4_ inhibitors that can reduce dietary energy loss. Dietary addition of monensin and NEOH decreased dry matter intake (*p* < 0.01); however, they increased the ADG of female lambs (*p* < 0.01). As a result, both monensin and NEOH increased feed conversion efficiency of the feedlotting lambs (*p* < 0.01), suggesting that feed energy saved from CH_4_ production promoted the feed efficiency and ADG in the present study. Except for the fact that NEOH addition increased the net muscle percentage to carcass weight (*p* = 0.03), neither monensin nor NEOH had a significant influence on carcass characteristics of female lambs (*p* > 0.05). From an economic point of view, NEOH and monensin caused a reduction in feed consumption costs, therefore resulting in a higher net revenue and economic efficiency than the control. In summary, dietary supplementation of NEOH in comparison with monensin presented a more promoting effect on energy utilization in female lambs by inhibiting rumen methanogenesis more efficiently, and NEOH improved the net revenue and economic efficiency more significantly than monensin.

## 1. Introduction

Ruminant herbivores depend on microbial fermentation within the rumen to acquire energy from plant compounds. Rumen function has been strongly manipulated by supplementing the forage diets with readily fermentable carbohydrates and additives in order to improve animal productivity [1,2]. However, this type of feeding sometimes induces rumen dysfunction because of an imbalance in the microbial populations [2]. Dietary supplementation of yeast cultures has been reported to increase body weight gain and feed conversion efficiency in livestock [3].

Methane (CH_4_) is an important greenhouse gas contributing to global warming [4,5]. Ruminants produce CH_4_ from fermentation, representing a loss of up to 12% of the feed energy [6,7]. A possible option to increase feed efficiency and hence ruminant productivity is to improve growth performance and feed conversion rate (FCR) of ruminant animals by decreasing ruminal CH_4_ production.

Monensin is a polyether ionophore that has been extensively used as a routine feed additive in ruminants to manipulate rumen fermentation to reduce methane production and increase feed efficiency [8]. Guan et al. [9] in an earlier study noted that monensin increased FCR by 6.3% in Angus yearling steers as a result of reducing the dry matter intake (DMI) without changing the average daily gain (ADG). Soltan et al. revealed that dietary supplementation of monensin in Barki lambs improved ADG and FCR by 25.2% and 29.2%, respectively [10]. In addition, numerous studies have reported significant reduction (6.5–12%) in CH_4_ emissions due to feeding monensin [11,12,13].

Several nitrocompounds, including nitroethane, 2-nitroethanol, 2-nitro-1-propanol and 3-nitro-1-propionic acid have been identified as reducing CH_4_ production both in vitro and in vivo [14,15,16,17]. Although some investigations have suggested that nitrocompounds such as nitroethane, 2-nitroethanol and 2-nitro-1-propanol do not have negative effects on ruminal fermentation or animal performance and the responses may differ depending on gender. In fact, a recent work by Zhang et al. compared the dietary addition effect of monensin and NEOH on ruminal methanogenesis and growth performance of male lambs [18], the latter one led to a greater improvement in ADG and FCR due to the lower CH_4_ production, but it is not clear if such a response could also occur in female lambs.

Previous studies illustrated that domestic farm animals differ in growth rate depending on their gender [19,20]. Abouheif et al. reported that the growth rate of Najdi ram lambs was higher than that of ewe lambs in the same age [21]. Aregheore also found that the males have a higher growth rate than females for the West African Dwarf goats [22]. However, the female lambs cannot be eliminated in feeding practice, especially in an intensive production system in China. How to improve the feed efficiency and animal performance of female lambs is also of great significance to increase the economic benefits for livestock producers. CH_4_ mitigation has the potential to improve feed energy efficiency; therefore, reducing CH_4_ production with NEOH and monensin may not only contribute to global efforts to reduce greenhouse gas emissions but could also improve the efficiency of feed conversion. In the present study, female lambs were served as experimental animals, and the objective was to evaluate the dietary supplemental effect of NEOH in comparison with monensin on nutrient digestibility, CH_4_ emission, growth performance and carcass characteristics as well as economic returns.

## 2. Materials and Methods

This study was approved by the Guidelines of the Beijing Municipal Council on Animal Care—Animal Production Ethics Committee (with protocol CAU20171014-1).

### 2.1. Chemicals

The light-yellow liquid nitroethanol (90% purity) was purchased from Beijing Lingrui Biotechnology Co., Ltd. (Beijing, China), and ionophore sodium monensin was purchased from Sigma Aldrich (St. Louis, Mo, USA).

### 2.2. Experiment Design and Animals Feeding

Sixty female, small-tailed Chinese Han lambs (29.6 ± 0.7 kg; 3.5 ± 0.3 month) were randomly assigned to one of three treatments: (1) a basal control diet (Control), (2) the basal diet supplemented with 40 mg/kg sodium monensin (monensin), (3) the basal diet supplemented with 277 mg/kg 2-nitroethanol (NEOH) on dry matter basis (DM). The dosage level of monensin and NEOH was in accordance with the reference of Soltan et al. [10] and Anderson et al. [14]. Lambs were housed with four animals to a pen (2 m × 5 m) with bamboo slatted floors. The pens were equipped with an individual feeding and watering troughs. All lambs were drenched against internal parasites and sprayed for ectoparasites prior to the experiment.

Diets were prepared as total mixed rations (TMR), which were formulated to meet their nutrient requirements (250 g/day) and growth requirements (Table 1). Lambs were adapted to their designated diet for 7 days before the experimental period. The whole feeding experiment consisted of the early stage (day 0–32) and later stage (day 33–70) of the fattening period. In order to avoid the adaptability of lambs to CH_4_ inhibitors, each stage included a treatment application period (day 0–16 and day 33–50) and a withdrawal of treatment period (day 17–32 and day 51–70). Sodium monensin and NEOH product were added in the treatment application period, while all three groups of lambs returned to be fed the same controlled diet in the withdrawal period. During the entire experiment, lambs had free access to water and mineral lick block.

### 2.3. Sampling and Measurements

The feed TMR was offered to lambs at 08:00 and 17:00. The amount of fresh TMR offered was adjusted according to feed intake of the previous day to ensure a 10% refusal. The feed offered and refused was weighted daily at per pen level, and samples were collected and ground through 1 mm screen, for subsequent analyses of DM. Dry matter intake (DMI) was calculated daily as the difference in dry matter between feed offered and the refusal. Live body weight of lambs was recorded before morning feeding, twice at the beginning and at the end of the experiment, respectively, and once every two weeks. Mean daily gain was calculated by the difference between two consecutive weightings. Feed conversion rate (FCR) was calculated as ADG divided by DMI every two weeks. Feed efficiency (FCR) was expressed as body weight gain per unit of feed consumption.

Feces samples of each animal were collected for 3 consecutive days in the morning at the end of each phase via grab sampling through rectal palpation. Feces samples were checked for gastrointestinal parasites by coprological analysis. Then, feces for the collection period were combined for each lamb and oven-dried at 65 °C over a four-day period. Dried feces were then pooled equally within pen for chemical analysis.

At the end of the experiment (day 70), 15 lambs from each treatment diet were randomly selected and their live body weight was recorded after fasting for 18 h. Lambs were then slaughtered by exsanguination using conventional humane procedures. Firstly, lambs were knocked unconscious using electroarcosis of 220V for 15 s. After bleeding, head, feet and skin were removed, and the carcasses were eviscerated. Their heart, liver, spleen, lungs, kidneys, digestive tract and the kidney–pelvic–gut fat were completely removed. The carcasses were subsequently weighed and chilled at 4 °C for 24 h. Then, carcasses were sawed into two symmetrical sides along backbone. After cutting the right side carcasses between the 12th and 13th ribs, the area of the longissimus dorsi was traced onto paper and the area was measured by computer scanning. The backfat thickness of the left side carcasses was determined over the deepest part of the loin-eye muscle. At last, the carcasses were separated and the net meat and bone were weighed. Dressing percentage was calculated as a ratio of fasting weight to carcass weight. Net meat percentage was calculated as a ratio of net meat weight to carcass weight.

### 2.4. Chemical Analyses

Samples of the consumed TMR, refusals and fecal matter were dried in a forced-air oven at 65 °C for 48 h and then ground to pass through a 1 mm screen. Following the procedures of AOAC (Association of Official Analytical Chemists, Gaithersburg, MD, USA), dry matter (DM; method ID 934.01), ash (method ID 942.05), crude protein (CP, method ID 976.06) and ether extract (EE, method ID 960.39) were analyzed [23]. According to the method of Van Soest et al. [24], neutral detergent fiber (aNDF) was determined with heat stable α-amylase and sodium sulfite addition and expressed inclusive of residual ash. Acid detergent fiber (ADF) were expressed inclusive of residual ash. Gross energy of diets and fecal samples were determined in an oxygen bomb calorimeter (MTZW–4, Shanghai Mitong Electromechanical Technology Co., Ltd., Shanghai, China).

### 2.5. Calculations

Body weight of each lamb (kg) at different feeding days (BW*d*) was fitted to a linear model Equation (1):

BW*_d_* = *i*BW_0_ + *k* × day (1)

where BW*_d_* is the body weight of lambs at dth feeding day; *i*BW_0_ is the initial body weight of lambs at day = 0; *k* is the coefficient factor (kg/day, ADG) in the linear model.

Nutrient digestibility was estimated by the indigestible acid insoluble ash (AIA) in diet and feces samples [25]. It was calculated as Equation (2):

Nutrient digestibility, % = [1 − (N_f_ × D_AIA_)/(N*_d_* × F_AIA_)] × 100% (2)

where, N_d_ and N_f_ is the nutrient concentration in diets and feces samples, respectively; D_AIA_ and F_AIA_ is the AIA concentration in diets and feces samples, respectively.

Gross energy intake (GEI), digestible energy (DE), metabolic energy (ME) and CH_4_ energy (CH_4_E) [26] was calculated as Equations (3)–(6):

GEI, MJ/d = gross energy × DMI;(3)

DE, MJ/d = GE digestibility × GEI;(4)

ME, MJ/d = 0.82 × DE;(5)

CH_4_E, MJ/d = 0.208 + 0.049 × GEI; (6)

### 2.6. Statistical Analysis

Data were subjected to ANOVA using the General Linear Model (GLM) procedure of SAS in which monensin and NEOH treatment was included as fixed effect. Least square means and standard errors of means were determined by LSMEANS procedure of the SAS. Tukey’s test was used for means comparison. Significance was declared at a level of *p* < 0.05 and a trend towards significance at *p* ≤ 0.10.

## 3. Results

### 3.1. Digestibility

Dietary addition of monensin and NEOH did not affect digestibility of DM, OM, CP, NDF and ADF of lambs (*p* > 0.05; Table 2).

### 3.2. Growth Performance

**Figure 1:**
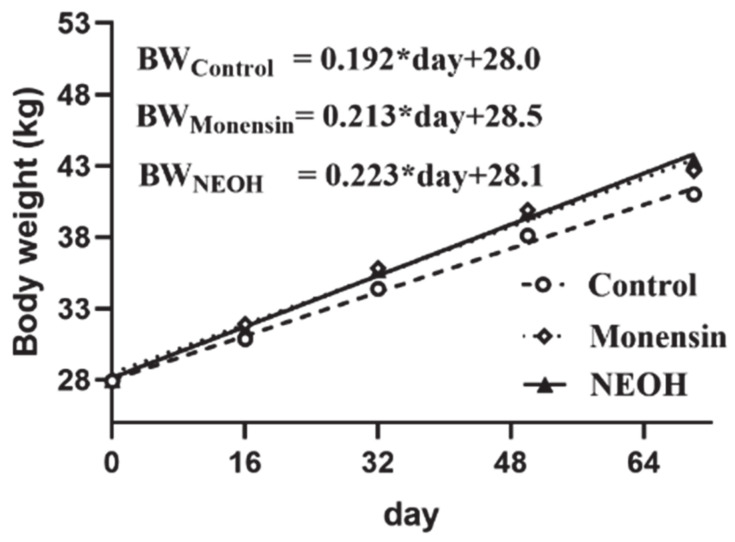
Dietary supplemental effects of monensin (40 mg/kg·DM) and 2-nitroethanol (NEOH, 277mg/kg·DM) on live body weight (BW) of female lambs.

The body weight (BW) of feedlotting lambs at different feeding periods was fitted to a linear model (Figure 1). The growth rate was greater in both monensin and NEOH group than in control group, and they ranked: NEOH > monensin > Control.

Dietary addition of monensin and NEOH decreased DMI (Table 3; *p* < 0.01); however, both monensin and NEOH increased ADG of female lambs (*p* < 0.01). As a result, the FCR of lambs was promoted by monensin and NEOH addition (*p* < 0.01).

### 3.3. Methane Emission

Both monensin and NEOH addition decreased the gross energy intake (GEI), digestible energy (DE), metabolizable energy (ME), and fecal energy loss of female lambs (Table 4; *p* < 0.01). Methane emission of fattening lambs was estimated based on DMI, GEI and DEI, and dietary addition of monensin and NEOH decreased CH_4_ emission (CH_4 DMI_, CH_4 GEI_ and CH_4 DEI_; *p* < 0.01) as well as methane emission per kg ADG (up to −12.7% vs. −17.4% decrease; *p* < 0.01). In addition, the NEOH in comparison with monensin exhibits greater inhibition on the CH_4_ energy (CH_4_E; *p* < 0.01). The ration of CH_4_E to GE in three groups ranked as NEOH (5.8) < monensin (6.0) < Control (6.3), and both monensin and NEOH decreased the ration of CH_4_E to DE and ME.

### 3.4. Carcass Characteristics

Except NEOH and monensin addition increased the net muscle percentage to carcass weight (%, Table 5; *p* = 0.03), neither monensin nor NEOH addition had a significant influence on carcass characteristics of female lambs (Table 5; *p* > 0.05).

### 3.5. Economic Evaluation

Dietary addition of monensin and NEOH reduced the feeding costs during the whole fattening period of female lambs by about CNY 2.7 and CNY 3.3, respectively. The economical evaluation of female lambs in the present study was conducted by two ways: sale benefit of live weight or sale benefit of carcass meat (Table 6). When evaluated by sale benefit of live weight: both monensin and NEOH were recorded the higher values in net revenue, economic feed efficiency and relative economic feed efficiency in comparison with control groups, and they ranked as NEOH > monensin > Control. However, neither monensin nor NEOH had an obvious effect on the economic evaluation when evaluated by sale benefit of carcass meat.

## 4. Discussion

It is well established that apparent total tract digestibility of nutrients was not influenced by monensin addition in feedlot heifers, lactating cows or growing lambs [10,27,28]. In the current study, apparent total tract digestibility of female lambs was not significantly influenced with monsensin and NEOH addition in the whole experiment period. Although NEOH is considered an efficient ruminal CH_4_ inhibitor in vitro [29,30], little information is available regarding its nutritive and feeding value as livestock feed. Only in a recent study of Zhang et al. [18], the supplementation of NEOH had no adverse influence on the apparent digestibility of nutrient. The DMI was decreased with monensin addition in the current study. In the previous studies, Bergen and Bates suggested that DMI was declined when monensin is added in diets [31]. Dietary monensin addition often has an ability to decrease rumen motility and dilution rate of digestion of nutrients, resulting in an increase of ruminal fill and consequently a reduction of DMI. Dietary supplementation of NEOH has recently been discovered to have a negative effect on DMI in fattening lambs [18]. Furthermore, in agreement with this study, the DMI was similarly decreased with NEOH addition in the present study. As an ionophore and growth promoter, monensin has been extensively used in commercial feedlot diets to increase feed efficiency and energy utilization [32,33]. Susin et al. reported that both feed efficiency and growth rate was promoted with monensin addition in lambs [34]. Safaei et al. and Tedeschi et al. found that the supplementation of monensin in grain-based feedlot diets improved feed efficiency by reducing DMI and had no effect on ADG [35,36]. In the current study, the ADG and feed efficiency of female lambs was also improved with monensin and NEOH supplementation. To our knowledge, both monensin and NEOH has a significant antimethanogenic activity in ruminants [10,14,17]. The reduction in CH_4_ production with monensin and NEOH supplementation, which represented less energy loss and increasing energy metabolism, may also improve feed efficiency of female lambs. Due to the requirement of a specialized methodology [37] and expensive equipment [38] in determination of CH_4_ production for individual ruminants, some empirical models have been developed to estimate specific CH_4_ emissions from ruminant animals [24,39,40]. Based on DMI, GEI and DEI, specific models for accurate estimation of CH_4_ emissions (CH_4 DMI_, CH_4 GEI_ and CH_4 DEI_) from sheep were obtained from Patra et al. [26]. In the present study, both monensin and NEOH addition reduced CH_4_ production, which involved CH_4 DMI_, CH_4 GEI_ and CH_4 DEI_. Monensin is a routinely used feed additive with the potential to decrease CH_4_ emissions from dairy cows, beef cattle and sheep. Odongo et al. observed obvious reductions in CH_4_ production (6.5–12% decrease) from dairy cows with dietary supplementation of monensin [12]. Vyas et al. evaluated the combined effects of monensin and 3-nitrooxypropanol on CH_4_ emissions of beef cattle, and found that the CH_4_ production was decreased by 26.9% when fed diet supplemented with monensin [13]. Recently, Soltan et al. also reported that monensin exhibited significant reductions in protozoal counts and CH_4_ emissions by 37.3% and 20.2% in growing lambs [10]. Until now, both in vitro and in vivo studies have demonstrated the antimethanogenic activity of nitrocompounds [10,14,17]. Anderson et al. [14] and Zhang et al. [41] reported that nitroethane and 2-nitroethanol were nearly equally effective in inhibiting ruminal in vitro CH_4_ production (up to 90% with 10 mM). However, in a recent study of Zhang et al., the CH_4_ production was reduced by up to 30% with NEOH addition in feedlotting lambs [18]. The antimethanogenic mechanism of NEOH in ruminants remains inconclusive, and further studies are needed to more fully elucidate the mode of action and toxicity of NEOH.

As reported by Eckard et al., farm managers are very interested in technologies which reduce methane production while increasing feed efficiency of bodyweight gain [42]. Fortunately, female lambs in the current study fed diet with monensin and NEOH produced less CH_4_ emissions per kg ADG (L/kg ADG) by 12.7% and 17.4% in comparison with the control, and feed efficiency, measured as FCR, was also improved. This phenomenon indicating that feed energy saved form CH_4_ production in monensin and NEOH-fed lambs promoted the feed efficiency and ADG in the present study. In comparison with the control, both monensin and NEOH decreased the percentage of CH_4_ energy in metabolizable energy. Therefore, the reduction in CH_4_ production may increase the efficiency of energy metabolism in monensin and NEOH groups.

Monensin is widely included in feedlotting diets of lambs to improve energy utilization and consequently promote feed efficiency [33]. The beneficial influence of monensin on energy utilization may attribute to its selective inhibition of Gram-positive bacteria (such as *Streptococus bovis*) that altered ruminal fermentation and increased propionate production [13]. Increasing rumen propionate production has the effect of increasing the efficiency of energy metabolism. Therefore, the reduction in CH_4_ production represented a less energy loss, thereby may result in the increasing efficiency of energy metabolism in female lambs.

Previous studies documented that the male animals usually showed a higher growth rate and fattening performance than females [20,21]; however, the female lambs cannot be eliminated in an intensive production system in China. In the present study, the average daily gain and feed conversion rate was improved by NEOH and monensin, but NEOH in comparison with monensin presented a greater promoting effect on energy utilization in the female feedlotting lambs by inhibiting rumen methanogenesis more efficiently. The improvement of feed efficiency and animal performance in female lambs is of great important to increase the economic benefits for livestock producers.

To our knowledge, these are the first published data evaluating the effect of dietary addition of NEOH on animal carcass characteristics. The basis of body weight gain of animals usually results from the increase in mass of the principal tissues, including muscle, bones and fat. In the present study, both NEOH and monensin promoted the net muscle percentage to carcass weight, suggesting that the carcasses were superior in monensin and NEOH-treated lambs than in control lambs. However, further studies are still needed in the future to evaluate the influence of monensin and NEOH addition on the carcass traits and non-carcass components. In an earlier study of Gilka et al. reported that carcass and meat quality were not affected by monensin in lambs [43]. In addition, Van Vuuren and Nel observed a lower proportion of meat but more fat in monensin-treated lambs [44]. In the present study, neither monensin nor NEOH had a negative effect on carcass characteristics of female lambs. However, the effect of dietary additive on food safety with respect to livestock product and animal health also needs future research.

Regarding economic evaluation, the economic efficiency was higher for lambs fed diets supplemented monensin and NEOH than control. Dietary addition of monensin and NEOH reduced the feeding costs during the whole fattening period of female lambs by about CNY 2.7 and CNY 3.3, respectively. However, both DMI and ADG were improved in monensin and NEOH group than in control, which will result in a higher net revenue and economic efficiency from long-term benefits.

## 5. Conclusions

In the present study, the average daily gain and feed conversion rate was promoted by NEOH and monensin, but NEOH in comparison with monensin presented a greater promoting effect on energy utilization in the female feedlotting lambs by inhibiting rumen methanogenesis more efficiently. NEOH and monensin increased the net muscle percentage to carcass weight, but neither NEOH nor monensin had an adverse effect on carcass characteristics. In addition, NEOH improved the net revenue and economic efficiency more significantly than monensin.

## Figures and Tables

**Table 1:** Ingredients and nutrient level of the total mixed ration (TMR) for feedlotting lambs.

Item	TMR
Ingredients (g/kg, as fed basis)	
Corn silage	600
Peanut vine hay	100
Corn meal	109.5
Wheat bran	30
Soybean meal	150
Limestone	3
Sodium bicarbonate	1.5
Salt	3
Premix ^1^	3
Chemical composition (g/kg, as dry matter)	
Organic matter	944
Crude protein (N×6.25)	163
Ether extract	22
Neutral detergent fiber	360
Acid detergent fiber	223
Calcium	5.5
Phosphorus	4.5
Gross Energy (MJ/kg)	15.2

^1^ The mineral-vitamin premix provided nutrients per kg of diet: Mn, 64 mg; Fe, 56 mg; Zn, 45 mg; Cu, 9.6 mg; Se, 0.3 mg; I, 1 mg; vitamin A, 48,000 IU; vitamin D, 11,000 IU; vitamin E, 33 IU; folic acid, 1.0 mg; nicotinic acid, 60 mg; d-calpanate, 30 mg and d-biotin.

**Table 2:** Dietary supplemental effects of monensin (40 mg/kg·DM) and 2-nitroethanol (NEOH, 277 mg/kg·DM) on total tract apparent digestibility of female lambs.

Treatment					
Item	Control	Monensin	NEOH	SEM	*p* Value
Dry matter digestibility, g/kg					
day 0~32	715	712	711	5.4	0.82
day 33~70	703	706	695	11.4	0.76
Organic matter digestibility, g/kg					
day 0~32	737	733	731	5.1	0.72
day 33~70	723	729	714	11.1	0.63
Crude protein digestibility, g/kg					
day 0~32	712	718	714	5.9	0.76
day 33~70	656	677	668	12.6	0.50
Neutral detergent fibre digestibility, g/kg					
day 0~32	577	563	552	8.7	0.19
day 33~70	560	543	482	30.3	0.23
Acid detergent fibre digestibility, g/kg					
day 0~32	558	567	541	22.0	0.72
day 33~70	552	570	561	37.8	0.94

SEM, standard error of the mean.

**Table 3:** Dietary supplemental effects of monensin (40 mg/kg·DM) and 2-nitroethanol (NEOH, 277 mg/kg·DM) on growth performance of female lambs.

Treatment					
Items	Control	Monensin	NEOH	SEM	*p* Value
Dry matter intake (kg/day)	1045 ^a^	998 ^b^	1006 ^b^	7.000	<0.01
Average daily gain (g/day)	186 ^b^	213 ^a^	218 ^a^	2.000	<0.01
Feed conversion ration	0.18 ^b^	0.21 ^a^	0.22 ^a^	0.003	<0.01

Means within a row with different letters differ (*p* < 0.05). SEM, standard error of the mean; feed conversion ration calculated as average daily gain divided by dry matter intake.

**Table 4:** Dietary supplemental effects of monensin (40 mg/kg·DM) and 2-nitroethanol (NEOH, 277 mg/kg·DM) on methane emission of female lambs.

	Treatment				
Items	Control	Monensin	NEOH	SEM	*p-*Value
Gross energy intake (MJ/day)	15.3 ^a^	14.6 ^c^	14.8 ^b^	0.05	<0.01
Fecal energy loss (MJ/day)	4.8 ^a^	4.6 ^b^	4.7 ^b^	0.04	<0.01
Digestible energy (MJ/day)	10.6 ^a^	10.3 ^b^	10.3 ^b^	0.03	<0.01
Metabolizable energy (MJ/day)	8.7 ^a^	8.5 ^b^	8.4 ^b^	0.02	<0.01
CH_4_ (L/day)	12.3 ^a^	11.3 ^b^	11.0 ^c^	0.03	<0.01
CH_4_ (L/kg ADG)	70.0 ^a^	61.1 ^b^	57.8 ^c^	0.80	<0.01
CH_4 DMI_ (L/day)	11.4 ^a^	11.1 ^b^	11.1^b^	0.02	<0.01
CH_4 DEI_ (L/day)	12.8 ^a^	12.6 ^b^	12.5 ^b^	0.02	<0.01
CH_4_E (CH_4_ energy, MJ/day)	0.96 ^a^	0.88 ^b^	0.86 ^c^	0.002	<0.01
CH_4_E in Gross energy (%)	6.3 ^a^	6.0 ^b^	5.8 ^c^	0.01	<0.01
CH_4_E in Digestible energy (%)	9.1 ^a^	8.5 ^b^	8.3 ^c^	0.02	<0.01
CH_4_E in Metabolizable energy (%)	11.2 ^a^	10.7 ^b^	10.3 ^c^	0.03	<0.01

Means within a row with different letters differ (*p* < 0.05). ADG, average daily gain; SEM, standard error of the mean.

**Table 5:** Dietary supplemental effects of monensin (40 mg/kg·DM) and 2-nitroethanol (NEOH, 277 mg/kg·DM) on carcass characteristics in female lambs.

	Treatment				
Items	Control	Monensin	NEOH	SEM	*p* Value
Live weight slaughtered, kg	43.6	44.2	42.0	0.65	0.07
Carcass weight, kg	21.5	21.1	20.4	0.49	0.35
Net muscle weight, kg	16.7	16.5	16.5	0.32	0.81
Net muscle, % carcass	77.7 ^b^	78.2 ^a^	79.3 ^a^	0.28	0.03
Bone weight, kg	3.5	3.6	3.5	0.07	0.73
Meat:bone ratio	4.8	4.7	4.7	0.13	0.61
Backfat thickness, mm	8.4	8.5	8.2	0.20	0.59
Lion eye area, cm^2^	21.7	22.3	21.3	1.26	0.85

Means within a row with different letters differ (*p* < 0.05). SEM, standard error of the mean.

**Table 6:** Economic evaluation of female lambs fed total mixed ration (TMR) supplemented with monensin (40 mg/kg·DM) and 2-nitroethanol (NEOH, 277 mg/kg·DM).

Items	CTR	MON	NEOH
Feed consumption, kg/lamb	121.5	117.0	116.0
Total feed cost, CNY/lamb ^1^	72.3	69.6	69.0
*Sale benefit of live weight* ^2^			
Live weight gain, kg/lamb	13.1	14.8	15.3
Income of gain, CNY/lamb	366	414	428
Net revenue, CNY/lamb	294	344	359
Economic feed efficiency	4.1	4.9	5.2
Relative economic feed efficiency (%)	100	120	127
*Sale benefit of carcass meat* ^3^			
Net meat mass, kg/lamb	16.4	16.5	16.6
Income of meat, CNY/lamb	951	957	963
Net revenue, CNY/lamb	879	887	894
Economic feed efficiency	12.2	12.7	12.9
Relative economic feed efficiency (%)	100	104	106

^1^ Total feed cost calculated on a price of 0.595 CNY/kg TMR in Chinese Yuan. ^2^ Economic benefits calculated on a market price of 28 CNY/kg live body weight in Chinese Yuan. ^3^ Economic benefits calculated on a market price of 58 CNY/kg lamb meat in Chinese Yuan; economic efficiency was calculated as the ratio between net revenue and total feed cost; relative economic feed efficiency, economic efficiency of additive-supplemented diet relative to the control diet.

## Data Availability

The data presented in this study are available on request from the corresponding author.
